# Anticancer Activity of Vitamin D, Lumisterol and Selected Derivatives against Human Malignant Melanoma Cell Lines

**DOI:** 10.3390/ijms252010914

**Published:** 2024-10-10

**Authors:** Paweł Domżalski, Anna Piotrowska, Robert C. Tuckey, Michał A. Zmijewski

**Affiliations:** 1 Department of Histology, Medical University of Gdansk, 1a Debinki, 80-211 Gdansk, Poland; paweldomzalski@gumed.edu.pl (P.D.); annapiotrowska@gumed.edu.pl (A.P.); 2 School of Molecular Sciences, University of Western Australia, Perth, WA 6009, Australia; robert.tuckey@uwa.edu.au

**Keywords:** vitamin D, 1,24,25(OH)_3_D_3_, lumisterol, melanoma, cytochrome P450scc, CYP11A1, VDR

## Abstract

Despite the recent development of improved methods of treating melanoma such as targeted therapy, immunotherapy or combined treatment, the number of new cases worldwide is increasing. It is well known that active metabolites of vitamin D_3_ and lumisterol (L_3_) exert photoprotective and antiproliferative effects on the skin, while UV radiation is a major environmental risk factor for melanoma. Thus, many natural metabolites and synthetic analogs of steroidal and secosteroidal molecules have been tested on various cancer cells and in animal models. In this study, we tested the anti-melanoma properties of several natural derivatives of vitamin D_3_ and L_3_ in comparison to 1,25-dihydroxyvitamin D_3_ (1,25(OH)_2_D_3_). A significant decrease in melanoma cell proliferation and cell mobility was observed for selected derivatives, with (25R)-27-hydroxyL_3_ showing the highest potency (lowest IC50) in A375 cells but lower potency in SK-MEL-28 cells, whereas the parent L_3_ failed to inhibit proliferation. The efficacy (% inhibition) by 1,24,25(OH)_3_D_3_ and 1,25(OH)_2_D_3_ were similar in both cell types. 1,25(OH)_2_D_3_ showed higher potency than 1,24,25(OH)_3_D_3_ in SK-MEL-28 cells, but lower potency in A375 cells for the inhibition of proliferation. As for 1,25(OH)_2_D_3_, but not the other derivatives tested, treatment of melanoma cells with 1,24,25(OH)_3_D_3_ markedly increased the expression of CYP24A1, enhanced translocation of the vitamin D receptor (VDR) from the cytoplasm to the nucleus and also decreased the expression of the proliferation marker Ki67. The effects of the other compounds tested were weaker and occurred only under certain conditions. Our data indicate that 1,24,25(OH)_3_D_3_, which has undergone the first step in 1,25(OH)_2_D_3_ inactivation by being hydroxylated at C24, still shows anti-melanoma properties, displaying higher potency than 1,25(OH)_2_D_3_ in SK-MEL-28 cells. Furthermore, hydroxylation increases the potency of some of the lumisterol hydroxy-derivatives, as in contrast to L_3_, (25*R*)-27(OH)L_3_ effectively inhibits proliferation and migration of the human malignant melanoma cell line A375.

## 1. Introduction

7-Dehydroholesterol (7DHC) is a key precursor of cholesterol; however, its exposure to ultraviolet B radiation (UVB) in the skin results in opening of the B-ring and formation of pre-vitamin D_3_ (pre-D_3_). Pre-D_3_ spontaneously isomerizes to vitamin D_3_ in a thermal, non-catalytic process. Exposure of pre-D_3_ to further high doses of UVB can result in resealing of the B-ring to form lumisterol (L_3_), or additional rearrangement of its double bounds to give tachysterol [1,2,3]. The classical pathway of vitamin D metabolism requires subsequent hydroxylation of vitamin D at C25 by CYP2R1 and at C1 by CYP27B1. The level of the major hormonally active form of vitamin D, 1,25(OH)_2_D_3_, is tightly regulated by its hydroxylation and inactivation by CYP24A1, whose gene expression is up-regulated by 1,25(OH)_2_D_3_ [4,5]. CYP24A1 is a mitochondrial cytochrome P450 that catalyzes the catabolism of 1,25(OH)_2_D_3_ predominantly by the C24 oxidations pathway with some contribution from the C23 pathway [6,7,8]. The initial product of the C24-oxidation pathway is 1,24,25(OH)_3_D_3_ and this accumulates due to its less efficient metabolism by CYP24A1 compared to 1,25(OH)_2_D_3_ [9]. Its subsequent metabolism by CYP24A1 eventually produces calcitroic acid which is excreted [5,7,8,10]. The C23-oxidation pathway through a series of hydroxylations and lactonization produces 1,25(OH)_2_D_3_-26,23-lactone [7,8]. Importantly, it is now clear that the key enzyme initiating steroidogenesis, CYP11A1 (also known as cytochrome P450scc), can also utilize vitamin D, lumisterol and tachysterol as substrates, producing series of biologically active hydroxy derivatives [8,11]. Key metabolites from vitamin D3 include 20(OH)D_3_ and 20,23(OH)_2_D_3_, and unlike cholesterol there is no cleavage of the side chain [12]. 20(OH)D3 can undergo hydroxylation at C24 by CYP24A1 producing 20,24(*R*)-dihydroxycholesterol (20,24(OH)_2_D_3_) [8]. CYP11A1 acts on L_3_ producing 22(OH)L_3_, 24(OH)L_3_ and 20,22(OH)_2_L_3_ while another CYP that acts on sterols, CYP27A1, produces 25(OH)L_3_ and the two C25 stereoisomers of 27(OH)L_3_, namely (25*R*)-27(OH)L_3_ and (25*S*)-27(OH)L_3_. CYP11A1-generated hydroxymetabolites of vitamin D and L_3_ and CYP27A-derived metabolites of L3 have been detected in human and animal sera and in some cases in skin and adrenal glands [13,14,15,16], with 20(OH)D_3_ and 1,20(OH)_2_D_3_ being present in human serum at substantially higher concentrations than 1,25(OH)_2_D_3_ [17].

In spite of its known anticancer properties, the use of 1,25(OH)_2_D_3_ in cancer therapy has been limited by its potential hypercalcemic effects [18]. Thus, for several decades many synthetic analogs of vitamin D and natural derivatives of vitamin D_3_, 7-DHC and lumisterol have been investigated and their anticancer properties tested on melanoma in vivo and in vitro [14,19,20,21,22,23,24]. Some of the resulting analogs displayed anti-proliferative, pro-differentiation and anti-inflammatory effects similar to or even better than 1,25(OH)_2_D_3_ [16,25,26]. Furthermore, vitamin D and its derivatives increase the anticancer properties of several antimelanoma drugs including doxorubicin, genistein, carboplatine [27,28,29,30], tamoxifen [31] and paclitaxel [32], both in vitro and using mouse models. Furthermore, the antitumor synergism between temozolomide and vitamin D on a model of glioblastoma was observed. The authors suggest that the combination of calcitriol, all-trans retinoic acid (ATRA) and temozolomide (known as CAT combination) can be a safe approach to benefit from vitamin D treatment of high-grade glial tumors [33]. It turns out that vitamin D also improves the anticancer properties of cisplatin and dacarbazine on melanoma cells [34]. This hormone not only improves the results of chemotherapy but also augments targeted therapy such as with cediranib where it was observed that 1,25(OH)_2_D_3_ enhances the anticancer properties of the Vascular Endothelial growth factor receptor (VEGFR) inhibitor [35]. Moreover, the same team discovered that vitamin D modulates the response of patient-derived metastatic melanoma cells to cediranib or vemurafenib therapy [36]. Another study showed that vitamin D sensitizes melanoma cells to a low dose of proton beam radiation, which suggests that it can be an adjuvant for this type of treatment of melanoma [37]. Interestingly, vitamin D and its analogs were shown to protect against UVB-induced damage in epidermal human keratinocytes and melanocytes [38]. Considering that UVB radiation is an important factor in the development of melanoma, vitamin D analogs may not only have application to anticancer therapy, but also to cancer prevention due to their protective effects against UVB. Additionally, low levels of 25(OH)D_3_ are associated with reduced survival in melanoma patients. A single nucleotide polymorphism in the vitamin D receptor affects development or progression of cancer and disease outcome further confirming the role of vitamin D in cancer [39].

In the current study, the effects of a range of natural hydroxy-derivatives of vitamin D3 and lumisterol were tested on melanoma cells and compared to those of 1,25(OH)_2_D_3_. Derivatives of vitamin D3 selected were 1α,24*R*,25(OH)_3_D_3_, which is the first major intermediate in 1,25(OH)_2_D_3_ inactivation produced by CYP24A1, and 20*S*,24*R*(OH)_2_D_3_, which is a product of the consecutive actions of CYP11A1 and CYP24A1 on vitamin D_3_, which has previously been shown to have biological activity, including on melanoma cells [40,41]. For L_3_, 22(OH)L_3_ which results from the action of CYP11A1 on L_3_ was selected to study as was (25*R*)-27(OH)L_3_ as a representative product of the action of CYP27A1 on L_3_. Both of these have previously been shown to exhibit biological activity on skin cells [11,14,16,19,38]. Two human malignant melanoma cell lines, A375 and SK-MEL-28, were selected to analyze the effects of these hydroxy-derivatives on cell proliferation and migration, cell cycle modulation, changes in the expression of selected genes and VDR translocation to the nucleus.

## 2. Results

### 2.1. The Hydroxy-Derivatives Were Synthesized Using Extracted and Purified CYP Enzymes Expressed in E.coli

The hydroxy-derivatives of vitamin D_3_ and L_3_ tested in this study, 1,24,25(OH)_3_D_3_, 20,24(OH)_2_D_3_, 22(OH)L_3_ and (25R)-27(OH)L_3_ (Figure S1), have all been detected in human serum [8,42], but their chemical synthesis is difficult [40] and they are not commercially available. However, CYP enzymes expressed in E. coli, extracted and purified, enabled us to utilize enzymatic synthesis to produce sufficient amounts (>300 nmol) of these sterols for testing their anti-melanoma activities on A375 and SK-MEL-28 cells.

### 2.2. The Hydroxy-Derivatives of Vitamin D_3_ and L_3_ Affects on Proliferation of Melanoma Cells A375 and SK-MEL-28

**Figure 1:**
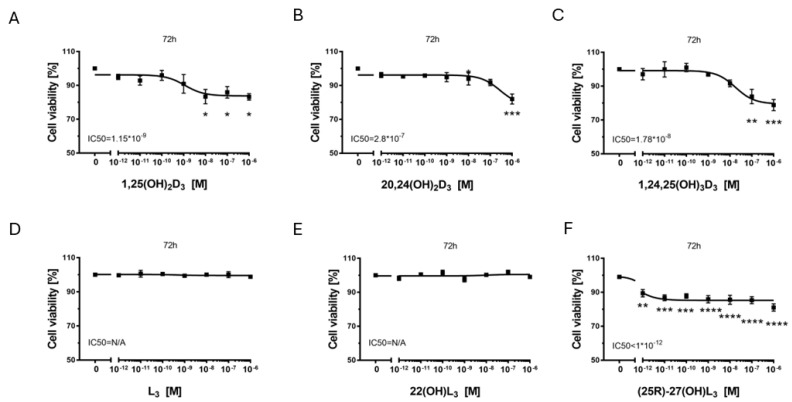
The effect of 1,25(OH)_2_D_3_, lumisterol and selected hydroxy-derivatives on the proliferation of human malignant melanoma A375 cells. Cells were treated with serial dilutions (10^−12^–10^−6^ M) of 1,25(OH)_2_D_3_ (**A**), 20,24(OH)_2_D_3_ (**B**), 1,24,25(OH)_3_D_3_ (**C**), L_3_ (**D**), 22(OH)L_3_ (**E**) or (25*R*)-27(OH)L_3_ (**F**) for 72 h and proliferation measured using the SRB assay. Data are shown as the mean from three independent experiments (*n* = 3) ±SD. Statistical significance was determined using One-Way Anova and presented as * *p* < 0.05, ** *p* < 0.005, *** *p* < 0.0005, **** *p* < 0.00005 vs. control.

**Figure 2:**
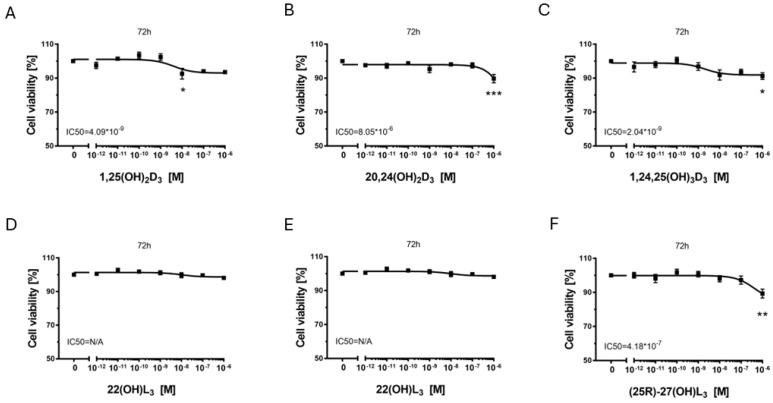
The effect of 1,25(OH)_2_D_3_, lumisterol and selected hydroxy-derivatives on the proliferation of human malignant melanoma SK-MEL-28 cells. The cells were treated with serial dilutions (10^−12^–10^−6^ M) of 1,25(OH)_2_D_3_ (**A**), 20,24(OH)_2_D_3_ (**B**), 1,24,25(OH)_3_D_3_ (**C**), L_3_ (**D**), 22(OH)L_3_ (**E**) or (25*R*)-27(OH)L_3_ (**F**) for 72 h and proliferation measured using the SRB assay. Data are shown as the mean from three independent experiments (*n* = 3) ±SD. Statistical significance was determined using OneWay ANOVA and presented as * *p* < 0.05, ** *p* < 0.005, *** *p* < 0.0005 vs. control; N/A—not available.

To assess the effect of these vitamin D and lumisterol derivatives in comparison to the well-characterized, hormonally active form of vitamin D, 1,25(OH)2D_3_, on the proliferation of melanoma A375 (Figure 1) and SK-MEL 28 cells (Figure 2), SRB assays were performed. All three vitamin D hydroxy-derivatives tested inhibited proliferation of melanoma A375 cells (Figure 1A–C). The IC50 value was the lowest (highest potency) for 1,25(OH)_2_D_3_ (1.15 nM), followed by 1,24,25(OH)_3_D_3_ (17.8 nM) and 20,24(OH)_2_D_3_ (280 nM). The highest inhibitory effect (efficacy), measured at the highest concentration of secosteroid tested (1 μM), was comparable for the 3 secosteroids (20–25%). The efficacy of the vitamin D hydroxy-derivatives for inhibition of proliferation was weaker in SK-MEL-28 melanoma cells, with only approximately 10% growth inhibition at the highest concentration (1 µM) of each derivative tested (Figure 2A–C). 1,24,25(OH)_3_D_3_ showed the highest potency with an IC50 half that of 1,25(OH)_2_D_3_.

For the lumisterols, neither L_3_ nor 22(OH)L_3_ inhibited proliferation of either cell type, with L_3_ actually causing a 10% increase in proliferation in SK-MEL-28 melanoma cells. In contrast, (25*R*)-27(OH)L_3_ (Figure 1F and Figure 2F) inhibited proliferation in both melanoma cell lines, with approximately 20% inhibition at the highest concentration tested (1 µM). High potency for this inhibition was seen with A375 cells (IC50 = 1 pM), but not for SK-MEL-28 cells.

### 2.3. The Hydroxy-Derivatives of Vitamin D_3_ and L_3_ Affects on the Mobility of Melanoma Cells A375 and SK-MEL-28

**Figure 3:**
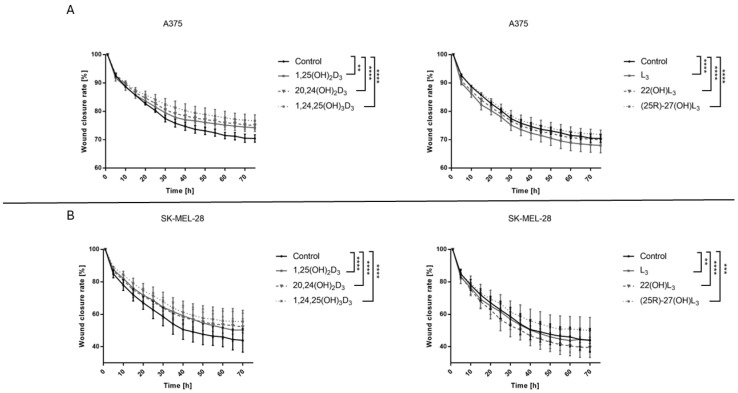
Effects of 1,25(OH)_2_D_3_, lumisterol and selected hydroxy-derivatives on the rate of a wound closure in A375 (**A**) and SK-MEL-28 (**B**) human malignant melanoma cells. After wounding, cells were treated with the hydroxy-derivatives (100 nM) and the cell-free area was measured every 30 min for 72 h using live imaging with the help of Olympus cell Vivo IX 83 software. The results were calculated as % wound closure. Statistical values (*n* = 3) were calculated with one-way analysis of variance and Tukey’s post hoc and presented as ** *p* < 0.01, *** *p* < 0.001, **** *p* < 0.0001.

The effects of the hydroxy-derivatives (100 nM) under study on the migration of A375 and SK-MEL-28 melanoma cells was examined. Interestingly, both melanoma cell lines differed in their migration efficiency with wound closure reaching ~30% for A375 and 50% for SK-MEL-28 cells; however, the effects of vitamin D and lumisterol derivatives were similar for both cell types (Figure 3). Treatment with 1,25(OH)_2_D_3_, 20,24(OH)_2_D_3_, 1,24,25(OH)_3_D_3_ or (25*R*)-27(OH)L_3_ resulted in slower migration of melanoma cells when compared to untreated cells, with statistical significance for both cells lines. 1,24,25(OH)_3_D_3_ decreased cell migration the most, leaving the wound at 75% of its original size for A375 cells and 40% for SK-MEL-28 cells. In contrast, L_3_ and 22(OH)L_3_ enhanced migration of A375 cells with the strongest effect being for L_3_ in comparison to untreated cells, while for SK-MEL-28 cells 22(OH)L_3_ increased migration more than L3. (Figure 3A,B). The representative pictures are shown in Figures S2 and S3.

### 2.4. The Hydroxy-Derivatives of Vitamin D_3_ and L_3_ Influence the Distribution of Cells in the Various Phases of the Cell Cycle

**Figure 4:**
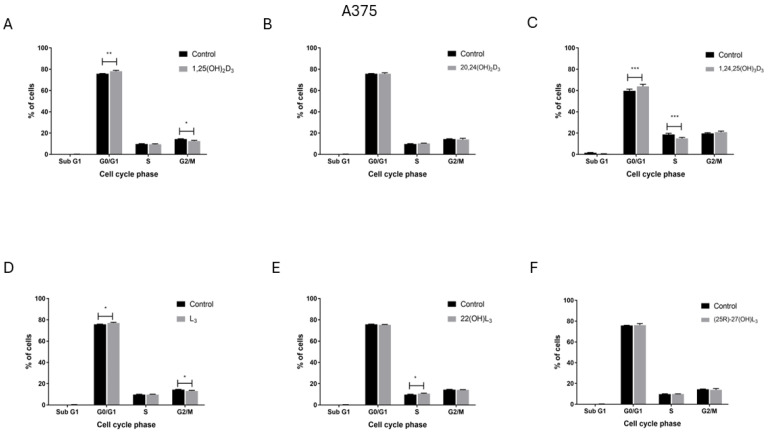
The effect of treatment with 1,25(OH)_2_D_3_, lumisterol and selected hydroxy-derivatives on the cell-cycle distribution of human malignant melanoma A375 cells. Cells were treated with sterols and secosteroids (100 nM) for 48 h, as indicated (**A**–**F**). Cells were harvested, stained with propidium iodide and the phases of the cell-cycle determined by Flow Cytometry (SubG1—apoptotic/necrotic cells, G1—growth, S—DNA synthesis, G2/M—preparation for mitosis). Data are presented as the mean ± SD (*n* = 3). Statistical significance was estimated using two-way Anova followed by Sidak’s multiple comparison test and presented as * *p* < 0.05, ** *p* < 0.01, *** *p* < 0.001. The Results are representative of three experiments.

**Figure 5:**
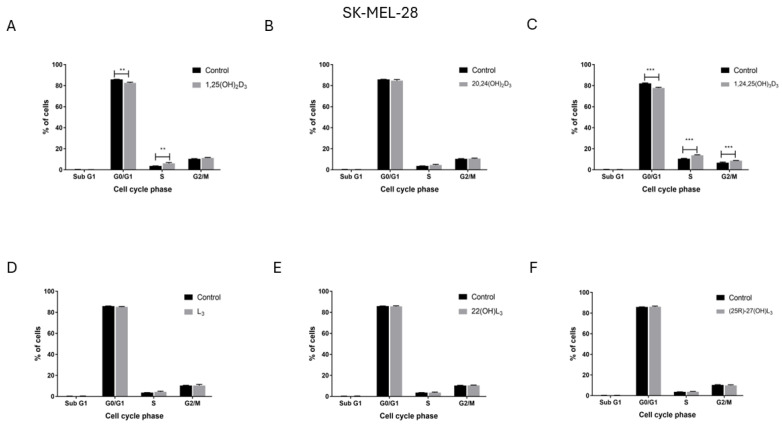
The effect of treatment with vitamin D, lumisterol and selected hydroxy-derivatives on the cell-cycle distribution of human malignant melanoma SK-MEL-28 cells. Cells were treated with sterols and secosteroids (100 nM) for 48 h, as indicated (**A**–**F**). Cells were harvested, stained with propidium iodide and the phases of the cell-cycle determined by Flow Cytometry (SubG1—apoptotic/necrotic cells, G1—growth, S—DNA synthesis, G2/M—preparation for mitosis). Data are presented as the mean ±SD (*n* = 3). Statistical significance was estimated using two-way Anova followed by Sidak’s multiple comparison test and presented as ** *p* < 0.01, *** *p* < 0.001. Results are representative of three experiments.

To explore the potential mechnisms of inhibition of A375 and SK-MEL-28 melanoma cell proliferation by the compounds under study, their influence on the distribution of melanoma cells in the various phases of the cell cycle was measured by flow cytometry. Treatment of the A375 melanoma cell line with 1,25(OH)_2_D_3_ (Figure 4A) or 1,24,25(OH)_3_D_3_ (Figure 4C) increased the percentage of cells in the G0/G1 phase of the cell cycle. The percentage of cells in the G2/M phase decreased after treatment with 1,25(OH)_2_D_3_ and in the S phase after treatment with 1,24,25(OH)_3_D_3_. For these cells L_3_ showed a similar but weaker effect to 1,25(OH)_2_D_3_ (Figure 4D) even though it did not inhibit cell proliferation (Figure 1 and Figure 2). In contrast, opposite effects to those seen on A375 cells were observed for SK-MEL-28 human melanoma cells following treatment. Both 1,25(OH)_2_D_3_ (Figure 5A) and 1,24,25(OH)_3_D_3_ (Figure 5C) increased the percentage of cells in G0/G1 phase and increased it in the S (1,25(OH)_2_D_3_) or G2/M and S (1,24,25(OH)_3_D_3_) phases. Neither 24,25(OH)_2_D_3_ nor L_3_ and its hydroxy-derivatives affected the cell cycle of SK-MEL-28 melanoma cells (Figure 5B,D–F). The representative flow fitting curve are shown in Figures S4 and S5.

### 2.5. The Hydroxy-Derivatives of Vitamin D_3_ and L_3_ Impact the Expression of Genes Involved in Vitamin D Metabolism

**Figure 6:**
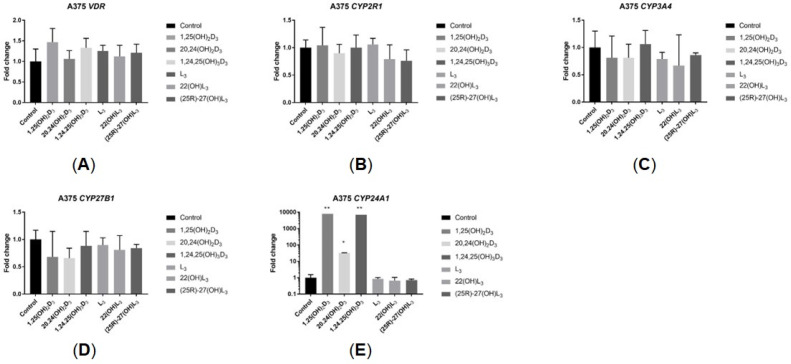
Effects of 1,25(OH)_2_D_3_, L_3_ and hydroxyderivatives on the expression of genes involved in vitamin D activation or metabolism in A375 human malignant melanoma cells. Cells were treated with the sterol or secosteroid (100 nM) for 24 h. mRNA levels were measured by qPCR. Data are shown as means ± S.D. of one representative experiment carried out in duplicate. * *p* < 0.05, ** *p* < 0.01 vs. control.

**Figure 7:**
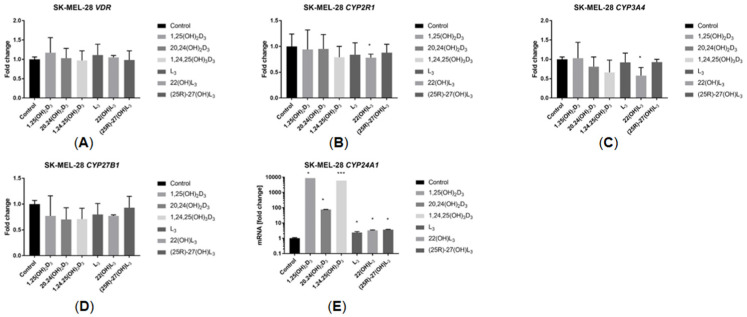
Effects of 1,25(OH)_2_D_3_, L_3_ and hydroxyderivatives on the expression of genes involved in vitamin D activation or metabolism in SK-MEL-28 human malignant melanoma cells. Cells were treated with sterol or secosteroid (100 nM) for 24 h. mRNA levels were measured by qPCR. Data are shown as means ± S.D. of one representative experiment carried out in duplicate. * *p* < 0.05, *** *p* < 0.001 vs. control.

The impact of the sterols under study on the expression of selected genes involved in vitamin D metabolism was investigated. As expected, 1,25(OH)_2_D_3_ dramatically increased the *CYP24A1* mRNA level in both cell lines. 1,24,25(OH)_3_D_3_ showed a similar effect, while 20,24(OH)_2_D_3_ caused a lower level of induction. L_3_ and its derivatives caused a small but significant increase in *CYP24A1* mRNA levels in SK-MEL-28 cells but not in A375 cells (Figure 6E and Figure 7E). The expression of other genes encoding CYPs that metabolize vitamin D, including *CYP2R1*, *CYP27B1* and *CYP3A4*, as well as the *VDR*, was not altered by the sterols under study (Figure 6A–D and Figure 7A–D).

### 2.6. 1,25(OH)_2_D_3_ and 1,24,25(OH)_3_D_3_ Influence VDR Translocation from the Cytoplasm to the Nucleus

**Figure 8:**
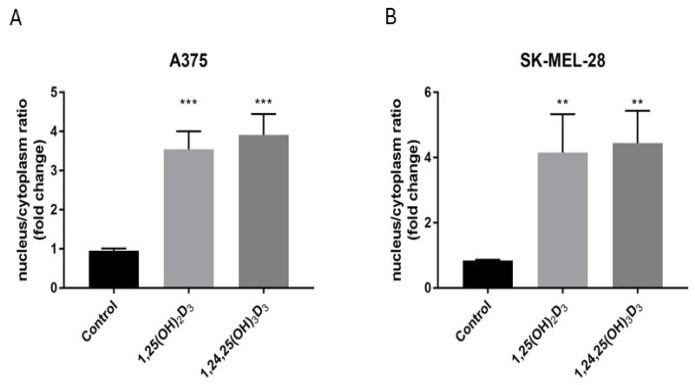
Effects of 1,25(OH)_2_D_3_ and 1,24,25(OH)_3_D_3_ on VDR translocation to the nucleus. A375 cells (**A**) or SK-MEL-28 cells (**B**) were incubated with the secosteroid (100 nM) for 1 h. The localization of the VDR was determined by immunostaining. Data are presented as the nucleus/cytoplasm ratio of the VDR (mean of fold change ± SD) calculated from the fluorescence intensities. The statistical significance was evaluated by the student t-test, ** *p* < 0.01, *** *p* < 0.001 vs. control.

Since at a concentration of 100 nM, 1,24,25(OH)_3_D_3_ showed comparable ability to 1,25(OH)_2_D_3_ to stimulate the expression of *CYP24A1*, we compared the ability of these two secosteroids to stimulate translocation of the VDR from the cytoplasm to the nucleus. The two sterols caused a similar 3- to 4-fold stimulation in the nuclear/cytoplasmic VDR ratio in both A375 and SK-MEL-28 cells (Figure 8A,B). The representative pictures are shown in Figures S6 and S7.

### 2.7. 1,25(OH)_2_D_3_ and 1,24,25(OH)_3_D_3_ Influence the Expression of the Ki67 Protein

**Figure 9:**
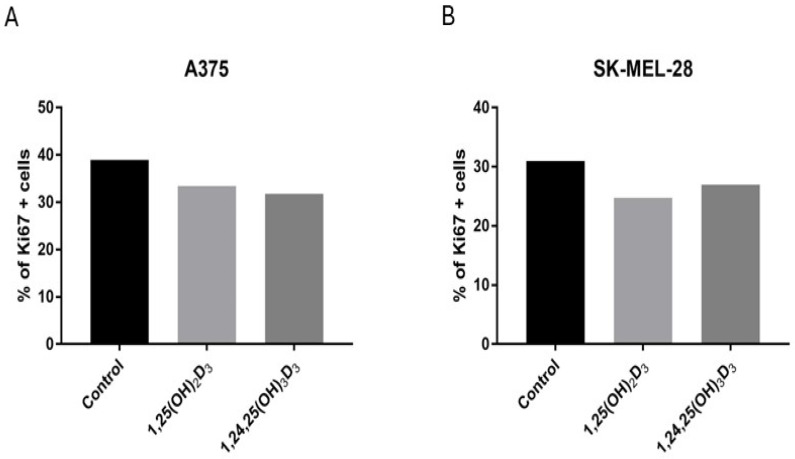
Effects of 1,25(OH)_2_D_3_ and 1,24,25(OH)_3_D_3_ on the expression of Ki67. A375 cells (**A**) or SK-MEL-28 cells (**B**) were incubated with the secosteroid (100 nM) for 1 h. Ki67 positive cells were determined by immunostaining. The fluorescence intensity threshold was set at the same level for each analyzed photo with yellow as an artificial color being applied to each cell above this cut-off. The number of these points was then counted using Olympus cellSens Dimension Deskop 4.3 software.

To complement the proliferation and cell cycle tests, the effects of 1,25(OH)_2_D_3_ and 1,24,25(OH)_3_D_3_ on the expression of the tumour proliferation marker Ki67 [43] were examined. These secosteroids were selected since they caused statistically significant effects on the inhibition of cell proliferation at a concentration of 100 nM in at least one cell line and caused the largest stimulation of *CYP24A1* expression. Treatment of both cell lines with 1,25(OH)_2_D_3_ or 1,24,25(OH)_3_D_3_ (Figure 9A,B) decreased the percentage of cells expressing Ki67. The effect was similar for both cell lines despite a much weaker response of SK-MEL-28 in the SRB test, with a stronger effect of 1,24,25(OH)_3_D_3_ for A375 and 1,25(OH)_2_D_3_ for SK-MEL-28 cells. The representative pictures are shown in Figure S8.

## 3. Discussion

Melanoma is the most common malignant skin cancer with a growing incident rate [44,45]. Several observational studies have demonstrated an association between higher serum vitamin D levels at the stage of diagnosis and better prognosis for melanoma patients [46,47,48,49]. It is well-established that the major hormonal form of vitamin D, 1,25(OH)_2_D_3_, activates the VDR, a nuclear steroid hormone receptor [50]. This receptor is found in many tissues, including the skin, where it is involved in photoprotection, which contributes to melanoma prevention [51,52,53]. 1,24,25(OH)_3_D_3_, the CYP24A1-derived metabolite of 1,25(OH)_2_D_3_, is considered as largely inactive being the initial product of 1,25(OH)_2_D_3_ oxidation and removal [8,54]. In contrast, the primary metabolite of CYP24 action on 20(OH)D_3_, namely 20,24(OH)_2_D_3_, has been reported to show increased anti-melanoma activity compared to the parent 20(OH)D_3_ [41]. The current study shows that both CYP24A1-derived metabolites, 20,24(OH)_2_D_3_ and 1,24,25(OH)_3_D_3_, inhibit the proliferation of human melanoma A375 cells maximally by about 25%, while for SK-MEL-28 cells this effect was markedly weaker. Interestingly, SK-MEL-28 cells are reported to have lower expression of the VDR than A375 cells [55]. We also found that the hydroxy-derivatives of vitamin D, including 1,25(OH)_2_D_3_, decreased the migration of cells in comparison to untreated cells and this effect was similar for both A375 and SK-MEL-28 cells. Furthermore, 1,24,25(OH)_3_D_3_ increased *CYP24A1* expression, a known VDR-driven genomic response [21], by the same degree as 1,25(OH)_2_D_3_. The effect was approximately 10-fold higher for A375 cells than SK-MEL-28 cells (fold change 10,000 vs 1000) which may be associated with the lower VDR expression in SK-MEL-28 cells, mentioned above. Stimulation of *CYP24A1* expression is by 1,24,25(OH)_3_ D_3_ is consistent with our results showing that it enhances VDR translocation to the nucleus to a similar degree to 1,24,25(OH)_3_D_3_.

1,24,25(OH)_3_D_3_, like 1,25(OH)_2_D_3_, also exerted anti-melanoma effects through increasing the proportion of cells in G0/G1 arrest and decreased the proportion in the S phase for A375 human malignant melanoma cells, with no effect on subG1 (an indicator of apoptosis/necrosis). Interestingly, the effect of 1,25(OH)_2_D_3_ on SK-MEL-28 was the opposite. This suggests the importance of the VDR for the anti-cancer activity of vitamin D hydroxy-derivatives since the level of tumor malignancy inversely correlates with VDR expression [56]. Thus, for the first time we have demonstrated the anti-cancer activity of 1,24,25(OH)_3_D_3_ using melanoma cell lines. In respect to the relative potency of 1,24,25(OH)_3_D_3_ compared 1,25(OH)_2_D_3_, for proliferation of A375 cells it was lower than for 1,25(OH)_2_D_3_ but for SK-MEL-28 cells it as two-fold higher, indicating a cell-type dependance. Previously 1,24,25(OH)_3_D_3_ was shown to stimulate HL-60 cells (a human leukemia cell line) to differentiate with a comparable potency to 1,25(OH)_2_D_3_ [8,57] despite showing a 2 to 3-fold lower affinity for the chick intestinal VDR [58].

Interestingly, although the expression of the proliferation marker Ki67 is dependent on the stage of the cell cycle [59], both 1,25(OH)_2_D_3_ and 1,24,25(OH)_3_D_3_ reduced the percentage of Ki67 positive cells in both cell lines. This is despite some differences in the effect of these two secosteroids on the distribution of cells at specific stages of the cell cycle. The anti-melanoma effects of 20,24(OH)_2_D_3_, which, unlike the other two secosteroids, tested lacks a 1α-hydroxyl group, was relatively weak, confirming the requirement for a 1α-hydroxyl group for strong interaction with the VDR [60].

In general, L_3_ and its hydroxy-derivatives, like vitamin D hydroxy-derivatives, demonstrate biological activity, including anticancer properties. This was further confirmed with the sterols tested in this work. In past studies, CYP11A1-derived 20(OH)L_3_, 22(OH)L_3_, 24(OH)L_3_ and 20,22(OH)L_3_ were shown inhibit the proliferation of human melanoma cells (SKMel-188) [16]. The effect was similar to that for the CYP11A1-derived hydroxyvitamin D_3_ analogs, but strong effects were confined to melanoma cells with no effect on normal melanocytes [16]. In addition, fibroblasts have been identified as a target for regulation by the hydroxylumisterols which act to inhibit basal and TNFα-induced NFκB transcriptional activity [16]. Hydroxylumisterols also stimulated translocation of the VDR from the cytoplasm to the nucleus, but the effect was weaker in comparison to 1,25(OH)_2_D_3_ and other hydroxyderivatives of vitamin D [16]. Despite this, CYP11A1-derived hydroxylumisterols do not appear to be involved in the regulation of genomic VDR activity [60]. The current study confirms the anti-cancer properties of hydroxylumisterols with CYP27A1-derived (25*R*)-27(OH)L_3_ inhibiting proliferation of A375 and SK-MEL-28 cells. Furthermore, in the case of A375 cells this effect occurred with an IC50 in the pM range. Surprisingly, the parent compound, L_3_, increased the proliferation of SK-MEL-28 cells by about 10%. We also show that L_3_ has similar properties to 1,25(OH)_2_D_3_ in terms of acting on the cell cycle, at least for A375 cells. It increased the proportion of cells in G1/G0 arrest while the proportion in G2/M arrest was decreased. One of the hydroxylumisterols tested, (25*R*)-27(OH)L_3_, slowed down cell migration for both A375 and SK-Mel-28 cell lines. It has been suggested recently that L_3_ derivatives may act through alternative receptors such as Liver X receptor (LXR), Retinoic Acid-related Orphan Receptor (ROR), and Aryl Hydrocarbon Receptor (AHR) [53,61].

In conclusion, despite recent advances in the treatment of melanoma and in light of emerging data of concern on the occurrence of resistance during treatment, it is prudent to continue to look for new treatment strategies. Vitamin D deficiency was shown to be one of the factors contributing to melanoma development [51,62] and might also have a negative effect on further therapy [63]. Despite the proven carcinogenetic effects of UV radiation, including on melanogenesis, it is also necessary for the synthesis of vitamin D_3_ in the skin [64,65]. Here, we show for the first time that 1,24,25(OH)_3_D_3_, the first metabolite in the catabolism of 1,25(OH)_2_D_3_, shows anticancer properties against melanoma cell lines generally similar to 1,25(OH)_2_D_3_, with cell-type dependent potency. In general, as shown for both D_3_ and L_3_ derivatives, hydroxylation increases their anticancer activity. However, the detailed mechanism of action of L_3_ derivatives remains to be established because they do not appear to work through the classical VDR-genomic pathway. Nevertheless, the naturally produced hydroxylated derivatives of vitamin D and lumisterol tested in this study, especially 1,24,25(OH)_3_D_3_ and (25*R*)-27(OH)L_3_, effectively inhibit proliferation and invasiveness of melanoma cell lines. This strongly supports the importance of vitamin D-related compounds in melanoma prevention, and the possibility of using them for melanoma treatment, especially as an adjuvant.

## 4. Materials and Methods

### 4.1. Synthesis of Hydroxy-Derivatives of Vitamin D_3_ and L_3_

1,24,25(OH)_3_D_3_ was produced by incubating 1,25(OH)_2_D_3_ with pure recombinant rat CYP24A1 and purifying the product by reverse-phase HPLC using both methanol in water and acetonitrile in water solvent systems, as described before [9]. It was produced in approximately 5% yield under the conditions employed with other intermediates of the C24-oxidation pathway also being products of the incubation. 20(OH)D_3_ was synthesized by incubating vitamin D_3_ with recombinant bovine CYP11A1. The HPLC-purified product was then incubated with recombinant rat CYP24A1 producing 20*S*,24*R*(OH)_2_D_3_. This was purified by HPLC using two solvent systems also as described before [66]. 22(OH)L_3_ was produced by the action of bovine CYP11A1 on L_3_ [15] while (25*R*)-27(OH)L_3_ was produced by the action of human CYP27A1 on L_3_ [14]. The structures of these products have previously been determined by mass spectrometry and NMR [14,40,41,66].

### 4.2. Cell Culture

Human malignant melanoma A375 and SK-MEL-28 cell lines were obtained from the American Type Culture Collection. The A375 cell line was cultured in DMEM supplemented with 10% fetal bovine serum and 1% penicillin/streptomycin while the SK-MEL-28 cells were cultured in MEM with 10% fetal bovine serum, 2 mM glutamine and 1% penicillin/streptomycin.

### 4.3. SRB Proliferation Assay

The sulforhodamine B (SRB) assay which measures protein content was performed as described previously [20,22]. Briefly, A375 and SK-MEL-28 cells were seeded in 96-well plates (3000 cells per well). Cells were cultured overnight and then subjected to treatment with a serial dilution of the hydroxy-derivatives being examined, for 72 h. After incubation, cells were fixed with 10% trichloroacetic acid (TCA) for 1 h at 4 °C. Medium was removed, plates washed with distilled water 5 times and left to dry overnight. Cells were stained with 0.4% sulphoroamide solution in acetic acid for 15 min. After staining, plates were washed with 1% acetic acid and air-dried. Finally, the SRB dye was solubilized with 10 mM Tris Base. Absorbance was measured at 570 nm using an Epoch spectrophotometer (BioTek, Wniooski, VT, USA).

### 4.4. Wound Healing Assay

Cells (A375 and SK-MEL-28) were seeded on an 8-well chamber slide at a density of 300,000 cells per well. After 24 h, the cells had formed a confluent monolayer. A mechanical wound was created by handmade scraping using a pipette tip. The hydroxy-derivatives to be tested were diluted in fresh medium at 100 nM concentration and then added to the chambers. The compounds being studied were used at a concentration of 100 nM, which corresponds to the optimal serum 25(OH)D3 level [67] to guarantee the maximum possible effect, although the plasma concentration of 1,25(OH)_2_D_3_ in humans is typically below 1 nM [48]. The migration of cells was observed for 72 h using a live imaging system with an Olympus cellVivo IX 83. The cell-free area was calculated as a percentage closure relative to the original size with cellSense Dimension Deskop 4.3 software (Olympus, Tokyo, Japan), as described before [35].

### 4.5. RT-PCR Assay of Gene Expression

Cells were seeded in 6-well plates at a destiny 150,000 per well. After 24 h, cells were treated with 100 nM of the hydroxy-derivatives to be tested. In another 24 h, cells were collected by using scrapers. Total RNA was isolated utilizing the ExtractME Total RNA Kit (Blirt, Gdańsk, Poland) following the protocol provided by the manufacturer. The concentration and purity of isolated RNA were determined using an Epoch Microplate Spectrophotometer (BioTek, Wniooski, VT, USA). Subsequently the RNA was reverse transcribed, and cDNA synthesized using a RevertAid First Strand cDNA Synthesis Kit (Thermo Fisher Scientific Inc., Waltham, MA, USA). The expression of selected genes was measured by Real-Time PCR and the primers used were described previously [34]. This was done using a StepOnePlus Real-Time PCR System (LifeTechnologies-Applied Biosystems, Grand Island, NE, USA) with RealTime AMPLIFYME SYBR Green No-ROX Mix (Blirt, Poland). *RPL37A* was used as a housekeeping gene to normalize expression of the genes under study [34].

### 4.6. Cell Cycle Analysis of Cells Treated with Hydroxy-Derivatives of Sterols and Secosteroids

Melanoma cells were seeded in 6-well plates at 150,000 per well. After 24 h of incubation, the medium was replaced with fresh medium containing 100 nM of the steroid hydroxy-derivative to be tested and the plates incubated for an additional 48 h. Cells were trypsynized and collected together with culture medium. Then, cells were fixed in 70% ethanol for 48 h and treated with ribonuclease in order to remove RNA. DNA was stained with propidium iodide for 30 min. The fluorescence of cells was measured by flow cytometry (FACSCalibur, Becton, Dickinson and Company, Franklin, Lakes, NJ, USA). The results were analyzed by CellQuest Pro software version 6.0 provided by Becton Dickinson. Data are presented as a percentage of cells with DNA content corresponding to the subG1 (apoptotic/necrotic cells) fraction or cells in G1, S and G2/M phases of the cycle.

### 4.7. VDR Translocation to the Nucleus

A375 and SK-MEL-28 cells were seeded on an 8-well chamber slide at a density of 10,000 per well. After 24 h 100 nM 1,25(OH)_2_D_3_ or 1,24,25(OH)_3_D_3_, diluted in a fresh medium, was added. Following a 1 h incubation, slides were washed 3 times for 5 min in PBS and fixed in 4% paraformaldehyde in PBS for 10 min. The cells were washed again (3 times for 5 min) and permeabilized for 5 min in 0.2% Triton X100 in PBS. Following another washing, slides were incubated for 30 min at RT with 1% BSA in PBS. After blocking, primary antibodies (anti-VDR, Santa Cruz Biotechnology, Dallas, TX, USA) were applied and cells were incubated at 4˚C overnight. The next day the primary antibody was decanted, and the slides were washed 3 times for 5 min in PBS, followed by incubation with secondary antibody (Alexa Fluor 488 anti-rabbit, Thermo Fisher Scientific Inc., Waltham, MA, USA) solution in PBS for 1 h at RT in the dark. After washing (3 times for 5 min in PBS), the slides were counterstained with 4′,6′-diamidinio-2-phenylindole (DAPI). Images were collected with an Olympus cellVivo IX83 and VDR translocation from cytoplasm to nucleus was determined using Olympus cellSens Dimension Deskop 4.3 software.

### 4.8. Ki67 Expression

A375 and SK-MEL-28 cells were seeded on an 8-well chamber slide at a density of 30,000 per well, incubated overnight, and then exposed to 100 nM 1,25(OH)_2_D_3_ or 1,24,25(OH)_3_D_3_, diluted in a fresh medium. Following a 1 h incubation, slides were washed 3 times for 5 min in PBS and fixed in 4% paraformaldehyde in PBS for 10 min. The cells were washed again (3 times for 5 min) and permeabilized for 5 min in 0.2% Triton X100 in PBS. Following another washing, slides were incubated for 30 min at RT with 1% BSA in PBS. After blocking, primary antibodies (anti-Ki67, Santa Cruz Biotechnology, Dallas, TX, USA) were applied and cells were incubated at 4 °C overnight. Following incubation, the cells were rinsed three times for 5 min in PBS, incubated with secondary antibody (Alexa Fluor 549 anti-mouse, Invitrogen, Carlsbad, CA, USA) solution in PBS for 1 h at RT in the dark, then rinsed three times for 5 min in PBS and incubated with DAPI. Images were collected with an Olympus cellVivo IX83 and the expression of Ki67 was determined using Olympus cellSens software.

## Data Availability

The data presented in this study are available on request from the corresponding author.

## Supplementary Materials

The following supporting information can be downloaded at: https://www.mdpi.com/article/10.3390/ijms252010914/s1.
