# Supplementary figures and images for: Anticancer Activity of Vitamin D, Lumisterol and Selected Derivatives against Human Malignant Melanoma Cell Lines
Source: Int J Mol Sci. 2024 Oct 10;25(20):10914. doi: 10.3390/ijms252010914 (PMC11506961; doi:10.3390/ijms252010914)

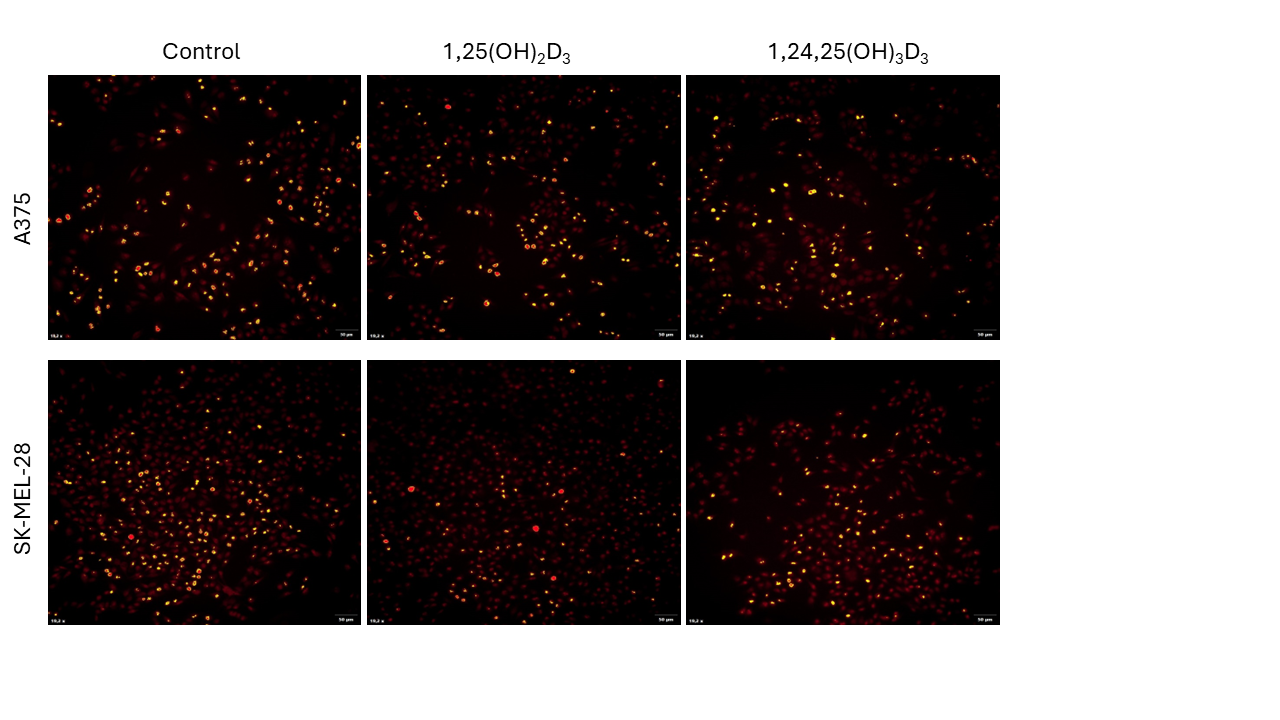

Supplement: Supplementary file 1 [file ijms-25-10914-s001.zip › Supplementary Figure 8..tif]

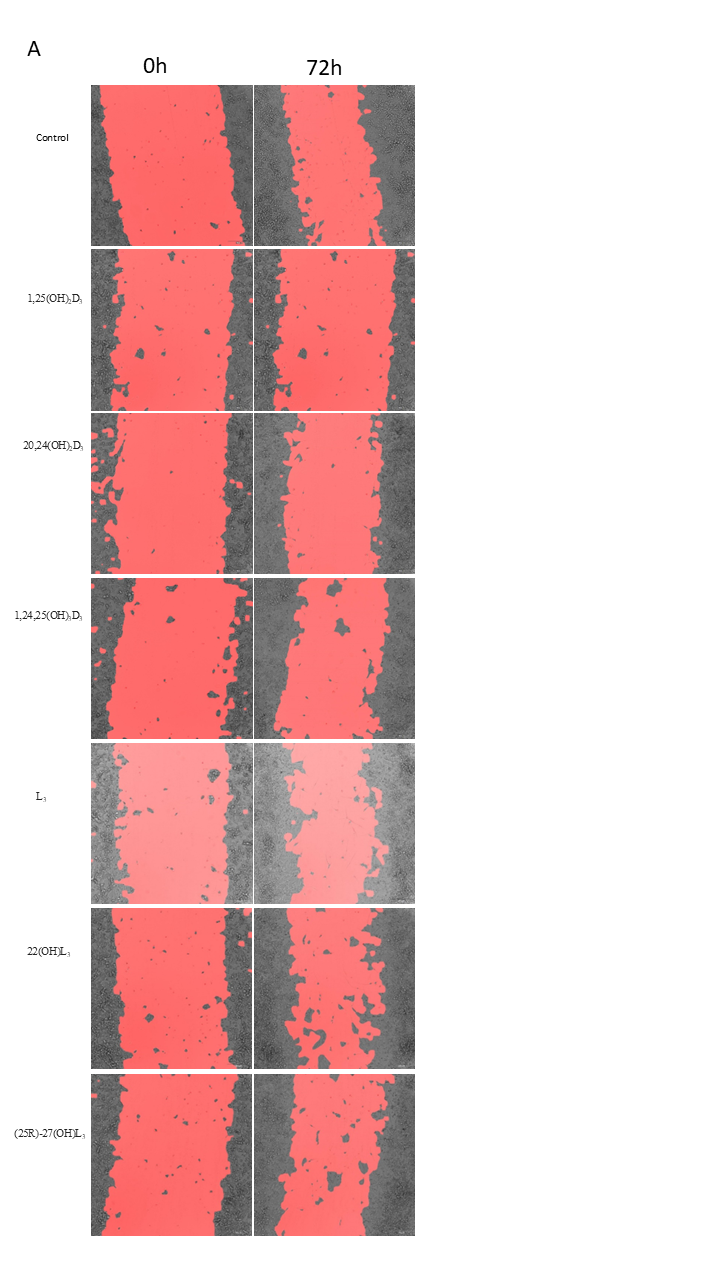

Supplement: Supplementary file 1 [file ijms-25-10914-s001.zip › Supplementary Figure 2..TIF]

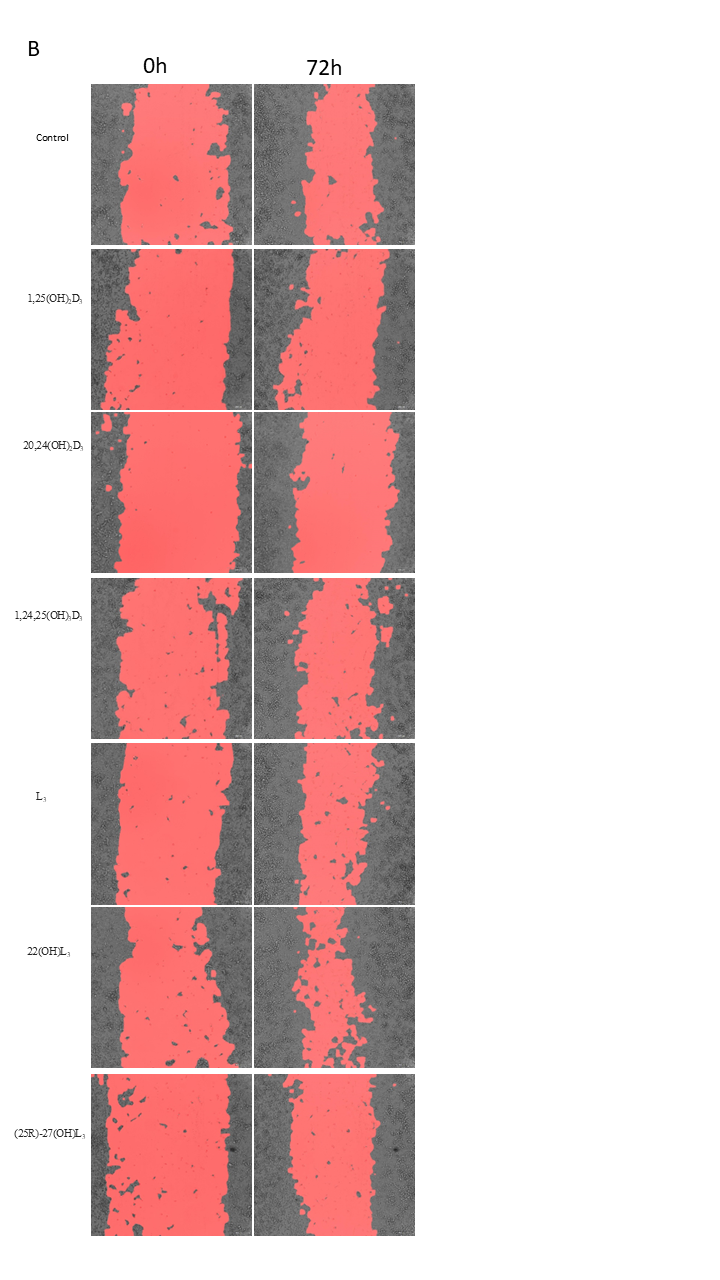

Supplement: Supplementary file 1 [file ijms-25-10914-s001.zip › Supplementary Figure 3..TIF]

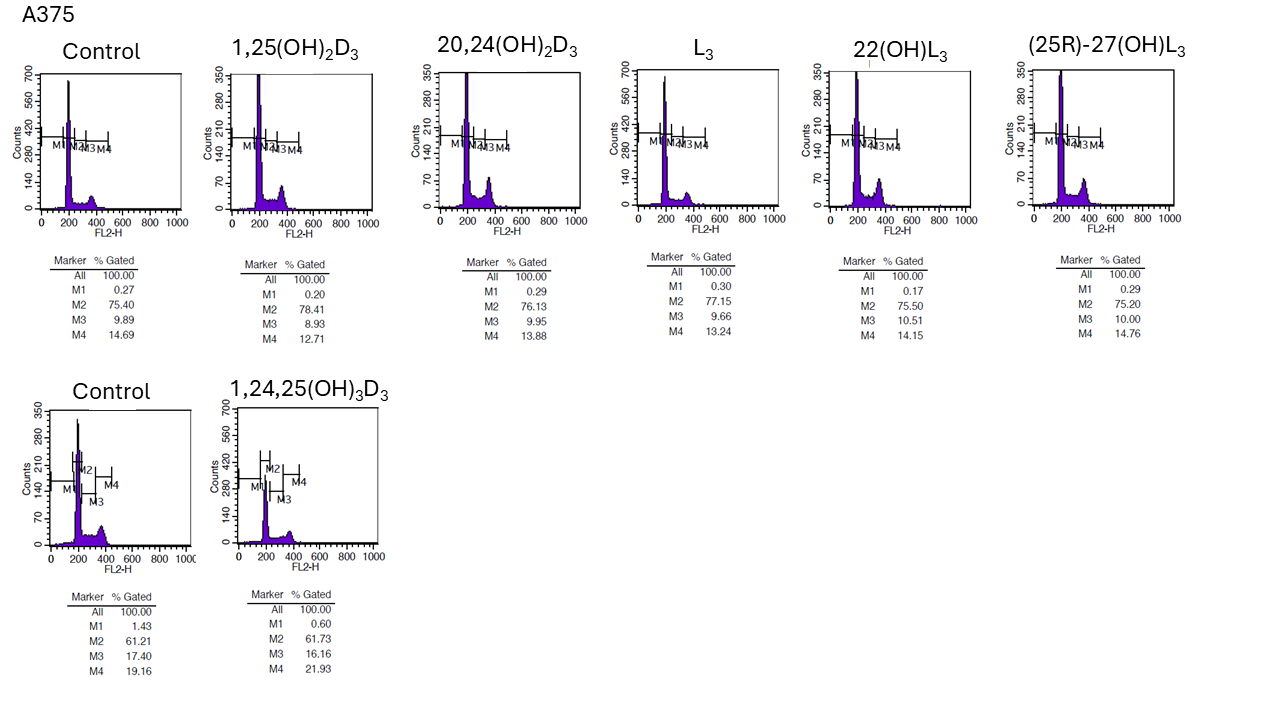

Supplement: Supplementary file 1 [file ijms-25-10914-s001.zip › supplementary Figure 4..tif]

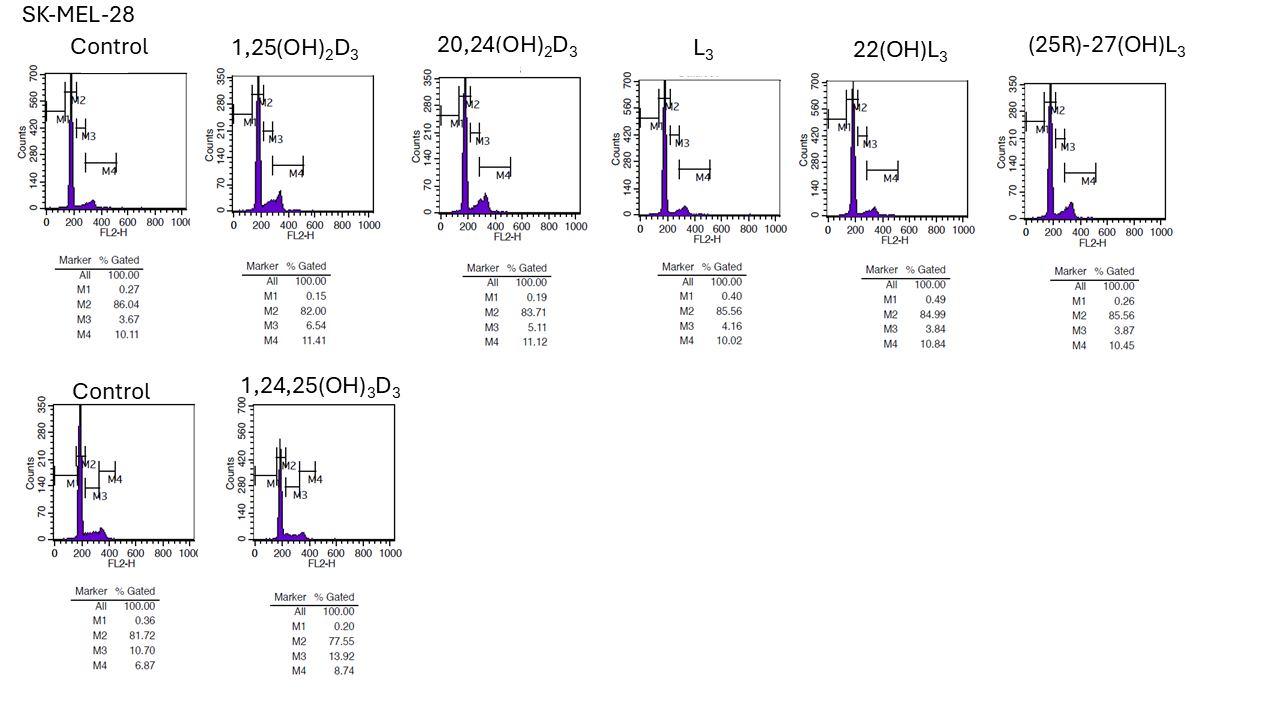

Supplement: Supplementary file 1 [file ijms-25-10914-s001.zip › supplementary Figure 5..tif]

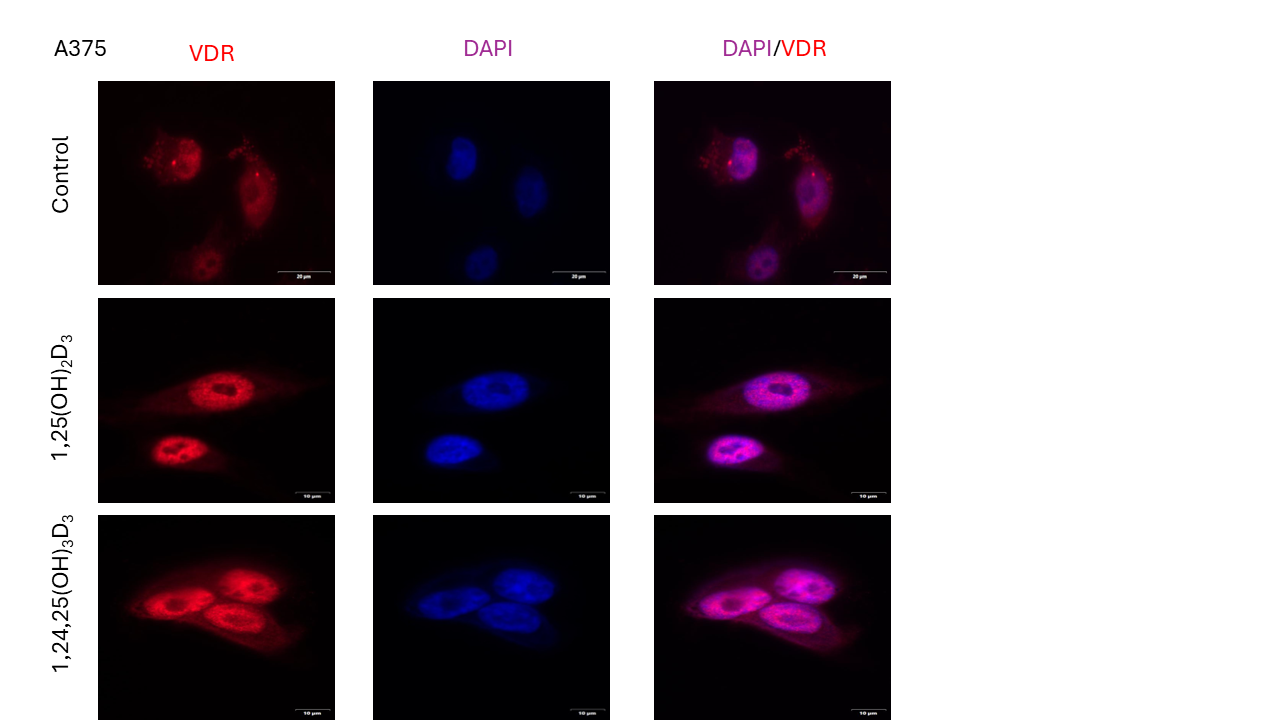

Supplement: Supplementary file 1 [file ijms-25-10914-s001.zip › Supplementary Figure 6..TIF]

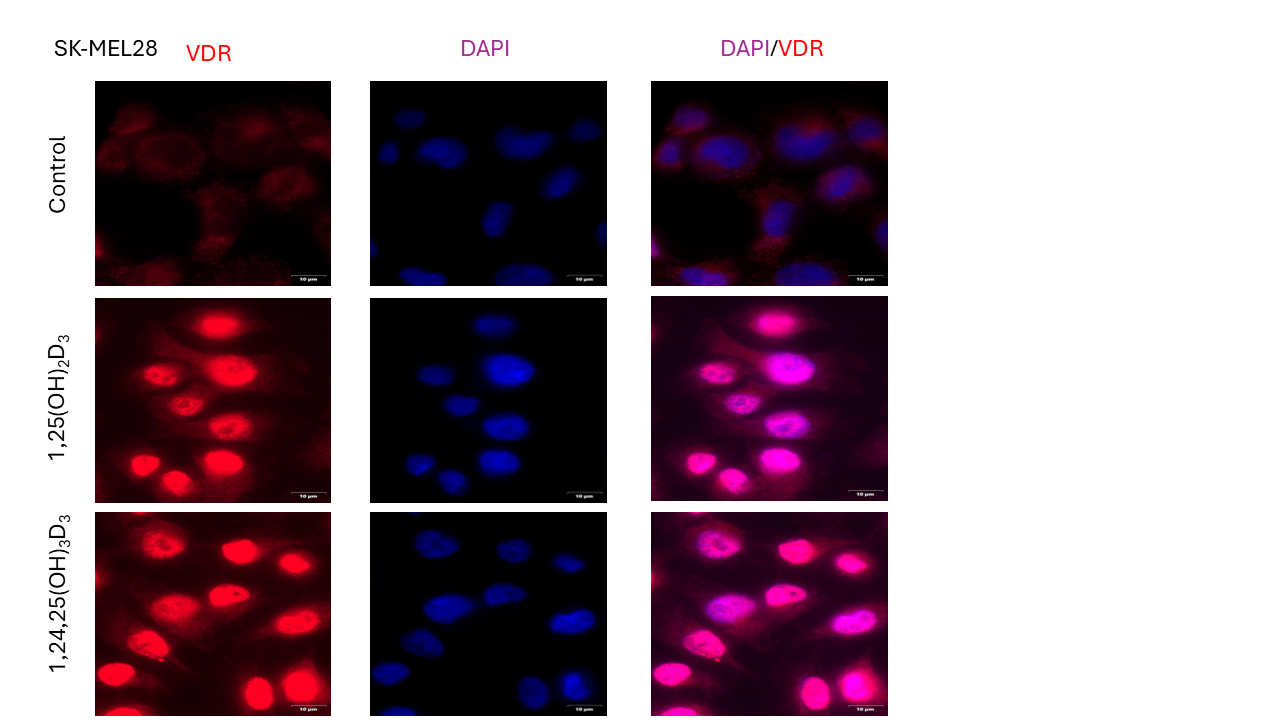

Supplement: Supplementary file 1 [file ijms-25-10914-s001.zip › Supplementary Figure 7.TIF]
